# Investigating the fluorescence in C-dots immobilised on alginate hydrogels-a study on diffusion kinetics and adsorption mechanisms†

**DOI:** 10.1039/d5ra01045d

**Published:** 2025-05-15

**Authors:** Jingyi Wang, Luz Karime Gil-Herrera, Ozge Akbulut, Ahu Gümrah Dumanli

**Affiliations:** a Department of Materials, The University of Manchester Oxford Road Manchester M13 9PL UK ahugumrah.parry@manchester.ac.uk; b Henry Royce Institute, The University of Manchester Oxford Road Manchester M13 9PL UK; c Faculty of Engineering and Natural Sciences, Sabanci University Istanbul 34956 Turkey

## Abstract

Immobilisation of fluorescent carbon dots (C-dots) in a hydrogel matrix, such as alginates, prevents fluorescence quenching in bioimaging and biosensing applications. However, critical parameters influencing the fluorescence, including the diffusion kinetics of C-dots and their distribution within the hydrogel matrix, remain unexplored. Herein, we investigated two distinct methods for immobilising C-dots within alginate hydrogel beads: (i) adsorption and (ii) premixing the C-dots prior to hydrogel cross-linking. Our batch adsorption experiments and kinetic model fittings revealed rapid, concentration-dependent diffusion from the external solution to the beads, along with their binding to surface active sites. The rate-determining step was the diffusion into interconnecting layers within the matrix, which impacts both diffusion rates and the overall distribution of C-dots within the beads. The fluorescence signal in the hydrogel matrix from the adsorption method exhibited limited penetration depth compared to the premixed method, which showed a more uniform distribution. We demonstrated that C-dots are well-immobilised and interact effectively with the hydrogel matrix, exhibiting stable fluorescence intensities and improved structural integrity. Our findings provide valuable insights into the interaction and diffusion of C-dots in hydrogel systems and will help advance research on the fluorescence properties of C-dots for bioimaging and bio-sensing applications.

## Abstract 2

Immobilisation of fluorescent carbon dots (C-dots) in a hydrogel matrix, such as alginates, prevents fluorescence quenching in bioimaging and biosensing applications.

## Graphical abstract

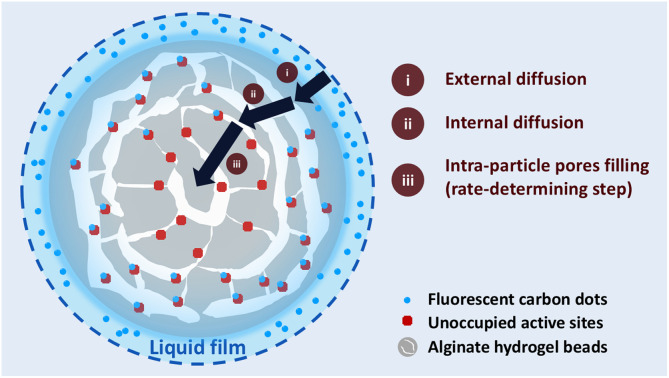

## 1. Introduction

In recent years, low-dimensional, small-scale carbon dots (C-dots) have received widespread attention from various fields due to their water solubility, low toxicity, biocompatibility, and especially tuneable photoluminescence (PL) properties and high photostability.^1–4^ Their outstanding fluorescence properties enable high sensitivity and selectivity detection based on the signals arising from the characteristic interactions of C-dots with anions,^5^ cations,^1,6^ organic matter,^7^ and bioactive substances.^8^ However, C-dots tend to self-quench and lose their fluorescence emission in the solid state due to particle aggregation.^9^ To overcome this, surface modification, such as preventing the graphitic cores from π–π interactions, has been suggested to control the interparticle spacing.^10^ Despite these efforts, unmodified C-dots struggle to maintain their excellent fluorescence properties outside aqueous environments, creating the need for scaffold materials that can effectively immobilise C-dots within particle carrier systems.^11^ Hydrogels, with their high water content and structural stability for *in vivo* and *in vitro* applications, are found to be particularly well-suited as carriers for C-dots, as they support both particle immobilisation and retention of fluorescence emission properties.^12,13^

Hydrogel carriers can be formulated with diverse chemical compositions, which impacts the functional behaviour of incorporated C-dots and, in turn, affects the overall performance of the system.^14^ Among different types of hydrogels used in bioimaging and biosensing, alginate hydrogels offer biocompatibility, hydrophilicity, and ease of gelation using simple ionic cross-linkers, making them one of the most widely studied hydrogel systems as particle carriers. Alginates are copolymers of β-d-mannuronic acid (M) and α-l-guluronic acid (G). The porous nature and surface functional groups (−COOH, –OH) make them an ideal carrier for the C-dots. Alginate can be readily cross-linked as the chains adopt a zigzag shape due to the composition of M and G structures, creating pocket-like cavities capable of easily accommodating Ca^2+^ ions, which is usually referred to as the egg-box model.^15^ Such alginate/fluorescent particle combinations have been extensively investigated for their potential use in drug delivery, *in vivo* bioimaging, tissue engineering, and the removal of pollutants like rare earth elements or heavy metal ions.^16–18^

While these studies showcased the functionality of C-dots immobilised in alginate hydrogel systems, there are several fundamental challenges arising from weak supramolecular interactions, and the limited distribution of the C-dots in the hydrogel matrix remains unexplored. Such issues can lead to the leaching of C-dots from the hydrogel networks and subsequently diminish the fluorescent response.^19^ Therefore, it is important to understand the interactions of the C-dots with the hydrogels, in particular their location after their immobilisation, which will influence the distribution of C-dots and their fluorescence efficiency. By correlating the structural and morphological properties of the alginate hydrogels with the fluorescence responses of C-dots, this work demonstrates the potential stability and distribution issues, thereby paving the way for optimising the performance of these systems. To elucidate such interactions, we investigated two district scenarios where (i) introducing C-dots into the alginate matrix *via* adsorption through diffusion from a suspension, and (ii) premixing the C-dots with the alginate solution prior to cross-linking. For this purpose, we synthesised N-doped C-dots and studied their diffusion and distribution in single-component systems (*i.e.*, the C-dots-loaded hydrogel beads were treated as a unified entity) to explore the interactions between the cross-linked structure and the abundant hydrophilic functional groups of alginates.

The batch adsorption studies were fitted to theoretical kinetic models with confocal imaging further validating these fittings by confirming the fluorescence distribution of C-dots throughout the alginate hydrogel. Our results showed clear differences in the localisation of C-dots with respect to the preparation methods; in scenario (i), where particles are adsorbed into the matrix, particle diffusivity towards the inner layers was limited. Meanwhile, in scenario (ii), a more homogeneous particle distribution was observed. We believe our work highlights that the diffusion dynamics of particles in hydrogel carrier systems depend on the method by which the particles are introduced into the hydrogel matrix. To the best of our knowledge, this is the first time an in-depth analysis combining the theoretical model fittings and traceable fluorescent microscopy was used to study the diffusion and distribution behaviour of fluorescent C-dots in the hydrogel structure.

## 2. Materials and methods

### 2.1 Materials

Citric acid (C_6_H_8_O_7_), urea (CO(NH_2_)_2_), alginic acid sodium salt (C_5_H_5_(OH)_4_O(COO)Na, *M*_w_ is between 120 000–190 000 g mol^−1^), and calcium chloride (CaCl_2_) were purchased from Sigma-Aldrich (UK). All chemicals were used without further purification. The dialysis tubing (molecular weight cut-off (MWCO 2000 Da)) was also purchased from Sigma-Aldrich (UK). All water used was purified to MilliQ standard, 18.2 MΩ cm (Select Fusion 320, Suez).

### 2.2 Synthesis of C-dots

A facile one-pot hydrothermal synthesis method was used to prepare C-dots by modifying the previous work by Vercelli *et al.*^20^ Specifically, 20 ml solutions of 0.2 M citric acid and 0.6 M urea in water were prepared as precursor solutions. This mixture solution was then transferred to a sealed 125 ml Teflon-lined stainless-steel autoclave and reacted at 160 °C for 4 h. The reaction suspension was cooled and centrifuged at 10 000 rpm to remove insoluble solid parts, and then purified using dialysis tubing (MWCO 2000 Da) for 24 h to remove unreacted low molecular weight precursors and by-products (*e.g.* possible fluorophores). The remaining C-dots solution was collected and freeze-dried (Labogene Scanvac CoolSafe Basic Freeze Dryer, Denmark) over 24 h to isolate the final solid-state products.

### 2.3 Preparation of ALG-as-made, ALG-C-dots-Ads, and ALG-C-dots-Hyb hydrogel beads

For the preparation of alginate hydrogel beads, freshly prepared sodium alginate solution in water (2 wt%) was bath-sonicated for 10 min to eliminate gas bubbles. Alginate hydrogel beads were prepared using a syringe pump (Ossila, UK) to extrude viscous alginate solution in a dropwise manner. Extrusion speed was kept at 1 μl s^−1^ through the attached Luer Lock PTFE Tubing (orifice diameter: 1.5 mm) and the droplets were released into a cross-linking bath containing 150 mM of CaCl_2_ solution under mild stirring. The obtained beads were separated using the filter paper, thoroughly rinsed by deionized water and then preserved in a deionized water bath until further use. Hydrogel beads that are prepared without any immobilisation of C-dots are labelled as ALG-as-made. The sizes of the ALG-as-made were determined by digital imaging of at least 50 beads to calculate the averages.

Two methods were used to load and immobilise the C-dots onto the alginate hydrogel beads. In the first method, the prepared pure alginate hydrogel beads (ALG-as-made) were added to a 50 mg per L C-dots solution to load the C-dots onto the beads through adsorption. The hydrogels prepared using this method was labelled as ALG-C-dots-Ads. In the second method, 50 mg L^−1^ of C-dots were premixed with 2% w/v sodium alginate to form a hybrid alginate-C-dots system. The mixture was then cross-linked in a 150 mM CaCl_2_ solution in a dropwise manner as described before. The hydrogel prepared using this method was labelled as ALG-C-dots-Hyb. In both cases, the volume of the aqueous sodium alginate solution and the quantity of C-dots in the integrated system were kept the same to ensure differences in fluorescent intensities resulted only from concentration changes caused by the immobilisation and dispersion of C-dots.

For the subsequent use of the hydrogel beads, excess surface moisture was removed from the wet samples using filter paper. To prepare the dried samples, the hydrogel beads were rapidly frozen using liquid nitrogen and then freeze-dried to maintain their porous structure and bead shape.

### 2.4 Characterization

Transmission electron microscopy (TEM, Talos F200X G2, operated at 200 kV, Thermo Fisher, USA) was used to analyse the morphology and size distribution of the synthesised C-dots. Image acquisition was carried out with Velox software. The particle size and size distribution were determined using ImageJ (v1.54i) by averaging more than 50 particles. The UV-visible absorbance spectra of the C-dots solution were recorded on a Lambda 365 UV-vis spectrophotometer (PerkinElmer, USA) in the 200–700 nm wavelength range. The fluorescence emission measurements were conducted on a Fluorolog-QM fluorimeter (Horiba Scientific, Japan) by exciting the C-dots solution with a wavelength of 320–400 nm using a slit width of 3 nm for incoming and outgoing beams and measuring emission up to 700 nm. Raman spectroscopy was performed on a LabRAM HR Evolution instrument (Horiba Scientific, Japan), equipped with a He–Ne laser (wavelength 633 nm), on the solid-state C-dots samples deposited on a silica wafer. A Fourier transform infrared spectrometer (FTIR, Bruker INVENIO, Germany) was used to determine the functional groups of synthesised C-dots, with a scanning range from 400 cm^−1^ to 4000 cm^−1^.

An AmScope SM-1 Series zoom binocular stereo microscope (AmScope, UK) was used to photograph the surface of the wet ALG-as-made beads. The beads diameter was determined using imageJ by averaging more than 50 beads. Scanning electron microscopy (SEM) was performed using a Zeiss Sigma VP FEG SEM system (Zeiss, Germany). Freeze-dried ALG-as-made samples were coated with an Au/Pt 80 : 20 target using a 50 mA current for 8 s with a Quorum Q150R Plus sputter-coater. The specific surface area and porosity of dried ALG-as-made beads were analysed using the Brauner–Emmett–Teller (BET) Quadrasorb EVO/SI automated surface area and pore size analyser (Quantachrome, USA).

Confocal microscopy images were taken of wet C-dots-loaded hydrogel beads deposited on glass slides using a Leica SP8 confocal microscope (Leica, Germany) with a 10 × 0.40 numerical aperture dry objective and a pulsed 405 nm laser for excitation. X-ray photoelectron spectroscopy (XPS) was performed using an Axis Ultra Hybrid spectrometer (Kratos Analytical, UK) with monochromated Al Kα radiation (1486.6 eV, 10 mA emission at 150 W, spot size 300 μm × 700 μm) with a base vacuum pressure of ∼5 × 10^−9^ mbar. Charge neutralization was achieved using a filament. Binding energy scale calibration was performed using C–C in the C 1s photoelectron peak at 284.8 eV. Analysis and curve fitting was performed using Voigt-approximation peaks using CasaXPS.^21^ Thermogravimetric analysis (TGA) and differential scanning calorimetry (DSC) were performed on the freeze-dried C-dots-loaded hydrogel beads. TGA was conducted using a SDT 650 instrument (TA Instruments, USA) under a nitrogen atmosphere from room temperature to 900 °C at a rate of 5 °C min^−1^. DSC was performed using a DSC 2500 instrument (TA Instruments, USA) with heating (0 °C to 200 °C), cooling (200 °C to 0 °C), and subsequent heating (0 °C to 400 °C) cycles at a rate of 10 °C min^−1^ under a nitrogen atmosphere. Values were presented from the second heating scan.

### 2.5 Adsorption performance experiments

The adsorption performance of C-dots onto alginate hydrogel beads was measured under the following protocols. The ALG-as-made wet beads ((1.01 ± 0.01) g per set) with excess surface moisture removed were added to 30 ml of C-dots solution at desired initial concentration gradients (20, 50, 80, 100 mg L^−1^) in amber screw-cap glass bottles. The pH of the initial C-dots solution was tested to ensure neutrality before adsorption. The amber bottles were fixed to an orbital shaker-incubator (ES-20, Grant Instruments, UK) at a constant temperature of 25 °C and a speed of 200 rpm. The ALG-as-made beads were treated in the C-dots solutions for various time intervals over a period of 28 hours. The remaining concentration of C-dots solution during adsorption was analysed by measuring the supernatant concentration using a UV-vis absorption spectrophotometer at an wavelength of 336 nm. Each batch was repeated three times under the same conditions.

Adsorption capacity *q*_t_ (mg g^−1^) or *q*_e_ (mg g^−1^) was calculated when adsorption reached at the certain and equilibrium time, respectively, using the following eqn (1) and (2):1
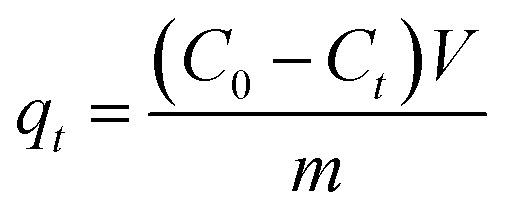
2
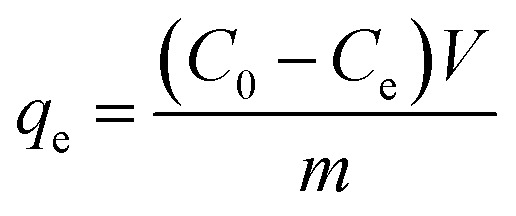
where *C*_0_, *C*_*t*_ and *C*_e_ (mg L^−1^) are C-dots concentrations at initial, certain time, and equilibrium time, respectively. *V* (L) represents for volume of initial solution and *m* (g) for mass of adsorbent.

To further analyse the cumulative adsorption of the C-dots, the removal efficiency factor (*R*, %) was calculated using the following eqn (3):3
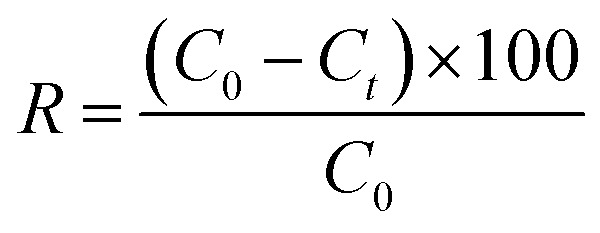


## 3. Results and discussion

**Scheme 1:**
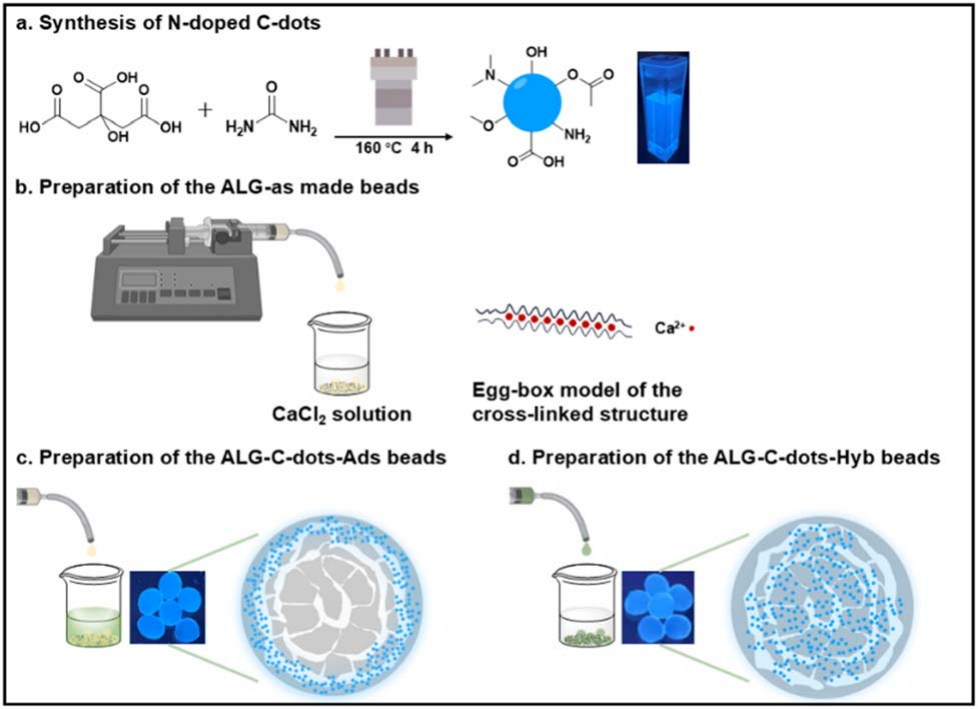
Schematic diagram of (a) the synthesis route of the fluorescent C-dots *via* the hydrothermal method; (b) the preparation of pure alginate hydrogel beads and the Ca^2+^-induced cross-linking, (c) and (d) two distinct scenarios investigated in this work for immobilising the fluorescent C-dots on alginate hydrogel systems, named ALG-C-dots-Ads and ALG-C-dots-Hyb, respectively, with different distributions of C-dots and fluorescence within the beads.

Scheme 1 represents the preparation steps and the predicted structures of the synthesised C-dots and cross-linked alginate hydrogel. The scheme illustrates the immobilisation method-dependent fluorescence distribution within the alginate beads.

### 3.1 Characterisation of C-dots

**Fig. 1:**
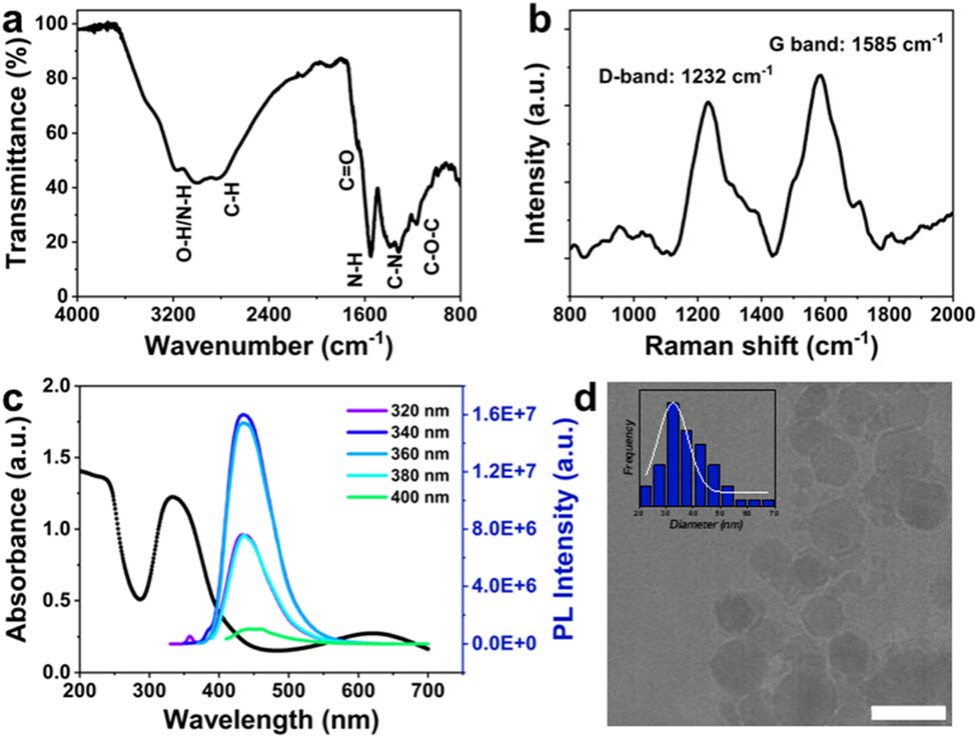
(a) FTIR, (b) Raman, and (c) UV-vis absorbance and PL intensity spectra of prepared C-dots; (d) TEM image (scale bar 50 nm) with size distribution of synthesised C-dots, the mean diameter of the C-dots was determined as (32.5 ± 13.0) nm, based on a fitted Gaussian model.

The surface functional groups of N-doped C-dots were investigated using FTIR spectroscopy (Fig. 1a) and XPS (Fig. S2†). The FTIR results confirmed the presence of O–H and N–H stretching vibrations between 2900 cm^−1^ and 3440 cm^−1^ indicating the presence of hydroxyl and amino groups that contribute to the highly hydrophilic nature of the synthesised C-dots. The band at 2840 cm^−1^ indicates the presence of an aliphatic C–H bond which is potentially from alkyl carbon stretching vibrations. The band at 1660 cm^−1^ with low intensity, the sharp band at 1550 cm^−1^, and the band at 1174 cm^−1^, are attributed to the C=O stretching of the carbonyl groups, N–H bending, and C–O–C stretching vibrations, respectively. The strong band observed at 1365 cm^−1^ was assigned to the C–N bond stretching, which is in alignment with the previous reports.^22^ Overall, the presence of these bands suggests the successful surface passivation of the synthesised C-dots.

The XPS spectrum in Fig. S2a† reveals that carbon, nitrogen, and oxygen are present on the surface of N-doped C-dots. In the core level scan spectra, the fitted C 1s peaks (Fig. S2b†) at 284.8 eV, 286.4 eV, 287.7 eV, and 288.8 eV can be assigned to carbon in the form of sp^2^ C bonding, C–O/C–N (sp^3^), C=O (sp^2^), and O–C=O (sp^2^), respectively. The fitted O 1s peaks (Fig. S2c†) at 531.5 eV and 533.1 eV are associated with oxygen in the states of C=O and C–OH/C–O–C, respectively. The fitted N 1s peaks (Fig. S2d†) at 400 and 401.5 eV indicate that the nitrogen exists in pyrrolic/amino N–H (sp^3^) and protonated pyridinic/graphitic-N (sp^2^) forms, respectively.^23–25^ The FTIR and XPS results correlate with each other, signifying that the synthesised C-dots have abundant surface functional groups with nitrogen atoms that were efficiently doped into the structure.

The Raman analysis supports the predicted structure of the synthesised C-dots. Two distinct D and G bands located at 1244 cm^−1^ and 1585 cm^−1^ can be attributed to sp^3^ defects (D-band) and sp^2^ carbon (G-band) in the C-dots structure, respectively, Fig. 1b.^26^ The ratio of peak intensities (*I*_D_/*I*_G_) is determined to be 0.86 for the synthesised C-dots, indicating that N-doped C-dots are highly graphitic.

The light absorption and fluorescence properties of the C-dots were investigated using UV-vis and fluorescence spectroscopy, as shown in Fig. 1c. The UV-vis spectrum shows two characteristic absorption peaks situated at 235 nm and 330 nm, corresponding to a π–π* transition of C=C graphitic core, and an n–π* transition of the functional bonds such as C–N/C=N/C=O.^27,28^ The PL intensity of the aqueous dispersion of C-dots exhibited clear excitation wavelength independence,^29^ displaying blue fluorescence emissions at 435 nm. As the excitation wavelength was changed from 320 nm to 400 nm, the PL intensity initially increased with the excitation wavelength, reaching its threshold at *λ*_ex_ = 340 nm, and then decreased. The fluorescence intensity and photostability of the synthesised C-dots confirmed their suitability for ensuring traceability in subsequent experiments. Although understanding the origin of the PL mechanism is not the primary focus of this study, the complex electronic energy structure of C-dots determines the various features of PL emission from their nanostructures.^29–31^ For the purposes of this work, we consider that the PL may arise from the distribution of emissive trap sites introduced by additional bonding states by introduction of nitrogen and oxygen into their structures.

### 3.2 Morphological analysis of alginate matrix hydrogels

**Fig. 2:**
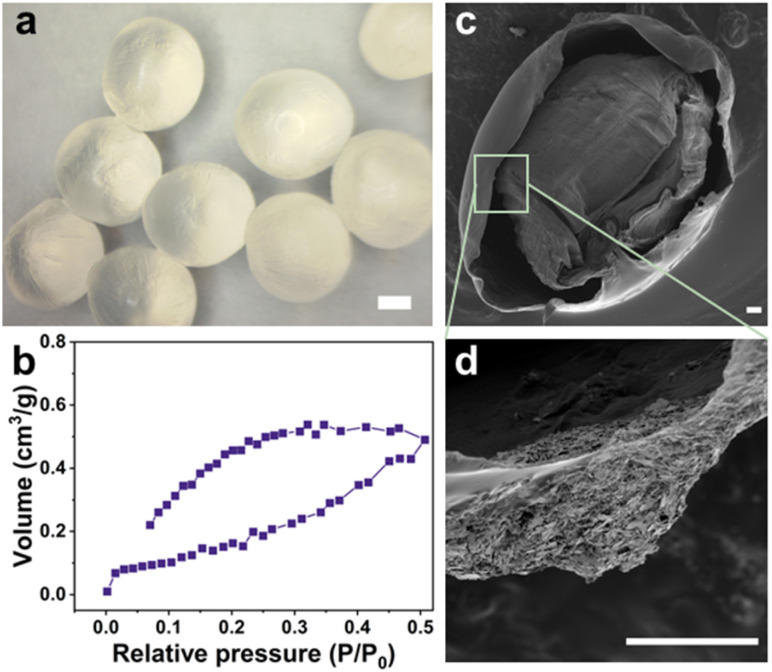
(a) Optical microscopy image (scale bar = 1 mm), and (b) N_2_ adsorption–desorption isotherms of the freeze-dried ALG-as-made beads; SEM images (scale bar = 100 μm) of (c) a cross-section and (d) a single inner layer of the freeze-dried beads.

The alginate hydrogel beads were produced *via* the dropwise spherification method into a coagulation/cross-linking bath. The hydrogel beads were found to have an average diameter of (3.75 ± 0.01) mm (Fig. 2a, and S3a†) which facilitated the ease of imaging and tracking of the fluorescence response.

The N_2_ adsorption–desorption isotherms of the ALG-as-made beads confirmed that the alginate network structure follows the type IV, indicating the presence of mesopores within the different layers.^32^ The surface area of the beads was measured as 1.181 m^2^ g^−1^, and the pore volume was 1.035 × 10^−3^ cm^3^ g^−1^ from the Barrett, Joyner & Halenda (BJH) method. These results indicate that the crosslinking process created a three-dimensional network with interconnected pores throughout the hydrogel structure. However, it is important to note that the rapid ionic crosslinking led to a non-uniform pore architecture, characterized by a denser, more tightly crosslinked outer layer and a more porous core. While the open network offers ease of particle diffusion, the core structure may not be fully accessible, potentially due to a distribution skewed towards smaller or less accessible pores.

The surface and cross-sectional SEM images (Fig. S3b,†2c and d) provided a detailed morphological structure of the cross-linked beads in their dry states. Upon freeze-drying, no severe cracks were observed, still, the alginate beads developed a wrinkled surface morphology suspected to be caused by the gelation and drying processes. The cross-sectional imaging analysis of the alginate beads revealed a similar multi-layered structure with discrete layers and some large cavities. Numerous mesopores, resulting from water entrapment during the gelation process and subsequent removal during freeze-drying, were present within the layers. The density gradient of the internal gel structure can be attributed to the diffusion kinetics of Ca^2+^ ions from the solution interacting with the outer layers to the inner layers.^33^ This results in a distribution gradient of alginate chains between the core of the droplets and the surface. Additionally, cross-linking initially occurs on the surface, forming a shell, this outer shell could potentially affect the continuous inward diffusion of Ca^2+^ ions into the beads and cause differences in the gelation of the alginate.^34,35^ Hence, the ALG-as-made beads have a porous core, from which concentric layers with highly open pores extend towards the shell, with channels interconnecting layers and a compact shell structure as described in Scheme 1c and d.

### 3.3 Adsorption performance of C-dots onto ALG-as-made beads

The three-dimensional porous alginate hydrogels are ideal hosts for the C-dots, as they can easily immobilise particles and facilitate the permeation and leaching processes.^36–38^ While such particle host–guest relation is desired for maintaining the fluorescence signal of the C-dots, the presence of several interfacial processes at the droplet surface and the core necessitates investigating the adsorption and diffusion properties of the ALG-C-dots system. Batch adsorption experiments of C-dots on ALG-as-made beads were carried out at neutral pH and 25 °C, over 28 hours to ensure the saturation of the C-dots within the hydrogel structure. The adsorption capacity and removal efficiency of ALG-as-made beads were examined to assess the advantages and limitations of the ALG-C-dots system for potential applications in bioimaging or biosensing against different contact times and initial C-dots concentrations.

#### 3.3.1 Effect of contact time on adsorption

To account for the interfacial processes between the C-dots and the alginate beads (*e.g.*, permeation, immobilisation, and leaching), a protocol was developed where ALG-as-made beads were added to a 100 mg per L C-dots solution. The adsorption effect was studied by analysing the UV-vis absorption intensities of the C-dots solution at different time intervals over 28 hours. A calibration curve (Fig. S4†) was established from the UV-vis absorption data to determine the concentrations at certain times and calculate the related adsorption capacity and removal efficiency.

**Fig. 3:**
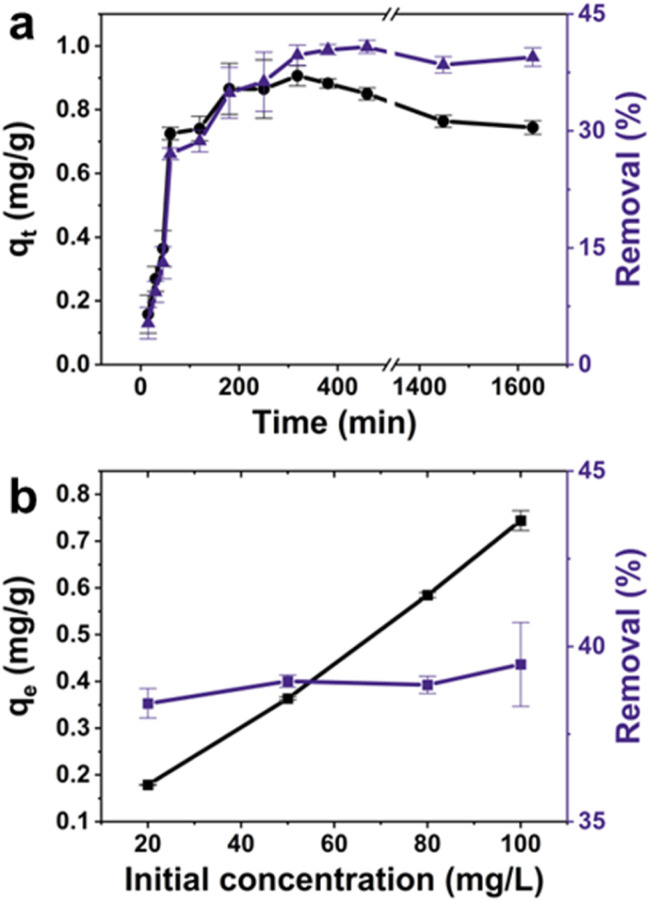
Effects of (a) contact time and (b) initial adsorbate concentration on the adsorption of fluorescent C-dots by ALG-as-made beads.

As shown in Fig. 3a, between 0 and 180 minutes, the adsorption capacity of C-dots experiences a rapid increase as the adsorption progresses, with the C-dots removal rate reaching (34.90 ± 3.28)%. Following this fast adsorption kinetics phase, the particle adsorption rate gradually decelerates, and the adsorption rate reaches a plateau between the time intervals of 180 and 460 minutes. It was confirmed that within this period, the adsorption capacity of the ALG-as-made beads was at (0.91 ± 0.03) mg g^−1^ with maximum adsorption and a maximum C-dots removal rate of (40.79 ± 0.84)%. The main reason for this initial fast adsorption and the subsequent plateau in the adsorption rate is attributed to the rapid combination of the C-dots with the available and unoccupied surface sites of the ALG-as-made beads. Subsequently, the C-dots slowly penetrated the interior of the adsorbents to occupy the active sites until there was no longer a driving force to capture the remaining C-dots in the solution. The adsorption capacity and removal efficiency both exhibit a slight decrease over a longer period of 1600 minutes. This could be caused by the fast adsorption phase, which could have potentially agglomerated some of the C-dots without immobilizing them on the hydrogel surface, leading to their potential leaching at later stages of the experiment.

#### 3.3.2 Effect of the initial concentration of C-dots solution on adsorption

The relationship between the initial concentration of C-dots and the adsorption efficiency was explored by preparing C-dots solutions at four different concentration values, ranging from 20 mg L^−1^ to 100 mg L^−1^. The adsorption experiments were conducted at a constant stirring speed of 200 rpm at 25 °C. As shown in Fig. 3b, as the initial concentration of the C-dots increased from 20 mg L^−1^ to 100 mg L^−1^, the adsorption capacity of the ALG-as-made beads for C-dots also increased, ranging from (0.180 ± 0.001) mg g^−1^ to (0.740 ± 0.020) mg g^−1^. This was consistent with the C-dots removal rate, which showed a modest increasing trend from (38.38 ± 0.42)% to (39.49 ± 1.20)%.

The initial concentration-dependent adsorption rates suggest the prepared ALG-as-made beads have abundant available sites on the surface, indicating a lack of a driving flow field as the concentration difference between the solution and the ALG-as-made beads decreases. Moreover, the relatively similar removal efficiency across different concentrations indicates that the adsorption process reaches a saturation point where the active sites on the bead surfaces become fully occupied. Once surface saturation is reached, additional C-dots in the solution have fewer sites to bind to, leading to the inner layers not being fully utilised and thus limiting the overall increase in removal efficiency. Therefore, as the initial C-dots concentration increases, the adsorption capacity increases, but the removal rate shows only a slight change.

### 3.4 Adsorption kinetic models and parameters

In complex processes such as adsorption/desorption of nanoparticles in hierarchically structured hydrogels, the thermodynamic analysis can only provide information about the final state of a system, whereas kinetics analysis can provide information on the process profile over time and is particularly concerned with the rates of changes in surface chemistry for practical applications.^39,40^ We explored the kinetics of the adsorption/desorption process to gain deeper insights into the rate-controlling steps. A 50 mg per L C-dots solution at neutral pH was adsorbed onto ALG-as-made beads at 25 °C to investigate the relationship between the adsorption capacity as a function of contact time. The experimental data were fit to kinetics models, including pseudo-first-order (PFO) kinetics, pseudo-second-order (PSO) kinetics, and intra-particle diffusion dynamics models. The equations of the PFO kinetics models eqn (4),^41^ PSO kinetics models eqn (5),^41^ and intra-particle diffusion dynamics models eqn (6),^42^ are as follows:

4 ln(*q*_e_ − *q*_*t*_) = ln *q*_e_ − *k*_1_*t*

5
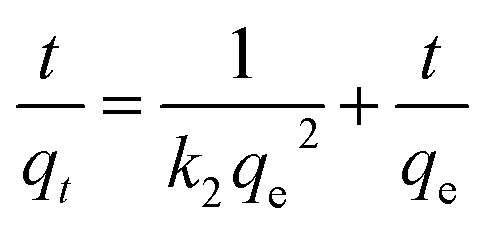

**Fig. 4:**
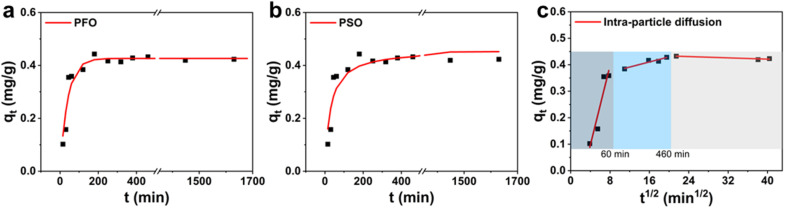
Kinetic analyses of fluorescent C-dots adsorption on ALG-as-made beads using (a) the PFO model, (b) the PSO model, and (c) the intra-particle diffusion model.

6 *q*_*t*_ = *k*_p_*t*^0.5^ + *C*

where *q*_e_ (mg g^−1^) and *q*_*t*_ (mg g^−1^) represent the adsorption capacity of C-dots per unit ALG-as-made beads during equilibrium at the time *t*, respectively. *k*_1_ (min^−1^), *k*_2_ (g mg^−1^ min^−1^), and *k*_p_ (g mg^−1^ min^−0.5^) are the rate constants of the PFO, PSO kinetics models, and the intra-particle diffusion dynamics models, respectively. The fitting coefficient *R*^2^ of the three models and theoretical adsorption amount *q*_e,cal_ of the models are given in Tables 1 and S1.† From the fitting results shown in Fig. 4a–c, the correlation coefficient *R*^2^ using the PFO model was found to be relatively high, and the *q*_e,cal_ value predicted by the PFO model agrees well with the experimental data *q*_e,exp_. Therefore, we concluded that the adsorption processes of C-dots on ALG-as-made beads were consistent with the PFO model. This indicated that high-concentration gradients dominate, driving particle migration toward the beads and the available surface sites.^43–45^ Compared with PSO kinetics,^46^ which are predominantly driven by pore filling of inner layers and active site interactions, the PFO model suggests that the adsorption/desorption process is initial concentration-dependent. Thus, the rapid initial uptake gradually slows down as the concentration gradient between the C-dots solution and the surface particle reaches equilibrium. This finding is consistent with the previously described adsorption performance and further enhances the understanding of the influence of bead morphology on the adsorption process.

Kinetic parameters for C-dots adsorption on ALG-as-made beads^a^

**Table:** 

Concentration (mg L^−1^)	*q*_e, exp_ (mg g^−1^)	Pseudo-first-order model			Pseudo-second-order model		
		*q*_e,cal_ (mg g^−1^)	*k*_1_ (min^−1^)	*R* ^2^	*q*_e,cal_ (mg g^−1^)	*k*_2_ (g mg^−1^ min^−1^)	*R* ^2^
50	0.4236	0.4266	0.0249	0.900	0.4598	0.0773	0.824

a *q*_e,exp_ (mg g^−1^) is the experimental value of adsorption capacity, *q*_e,cal_ (mg g^−1^) is the theoretical value of adsorption capacity.

**Table:** 

Concentration (mg L^−1^)	Intra-particle diffusion model			
	Step	*k*_p_ (g mg^−1^ min^−0.5^)	*C*	*R* ^2^
50	1	0.075	−0.2026	0.8846
	2	0.0048	0.3337	0.9094
	3	0.0006	0.4455	0.8416

In addition, the intra-particle diffusion model, which describes the kinetics of adsorption where the rate-limiting step involves the diffusion of the adsorbate particles within the porous adsorbent material, while the diffusion of the adsorbate in the liquid film around the adsorbent and the adsorption onto the active sites are instantaneous.^45,47,48^ The fitting results demonstrated three linear trends, with respective *R*^2^ values of 0.88, 0.91, and 0.84 (Table 1). These *R*^2^ values suggest that the kinetics of C-dots adsorption into alginate hydrogel beads follow a multi-stage process, with each stage involving different mass transfer mechanisms. As shown in Fig. 4c, within the first 60 minutes (indigo-shaded), there was a relatively efficient adsorption process, involving surface adsorption or fast external surface adsorption. This was followed by a transition phase at 460 minutes (blue-shaded), likely influenced by both external mass transfer and the onset of intra-particle diffusion into the pores of the hydrogel beads. During this phase, the adsorption rate slows as surface sites become gradually occupied and diffusion into the pores becomes more significant. The third stage (grey-shaded) is likely the slowest and may represent the final equilibrium stage, where intra-particle diffusion becomes the dominant mechanism. The constants (intercepts), which are closely related to the boundary layer effect,^42^ increased across the three linear fittings. This increase suggests that the boundary layer effect becomes more significant as the adsorption process progresses.

### 3.5 Fluorescence distribution of the prepared ALG-C-dots systems

Confocal microscopy results provided detailed insights into how the C-dots interact with the hydrogel matrix, using visual cues to support the theoretical adsorption models. To identify the particle distributions within the hydrogel beads, the ALG-C-dots-Ads and ALG-C-dots-Hyb samples were removed from the C-dots solutions and gently blotted to eliminate excess surface moisture. The hydrogel beads were manually cut using a clean blade to acquire their cross-sectional images. Samples were then mounted directly on cleaned glass slides and imaged.

**Fig. 5:**
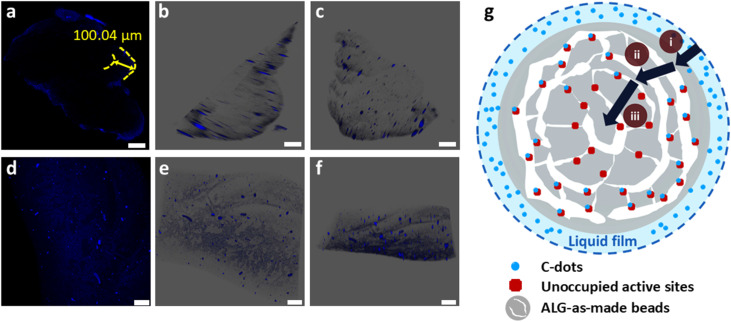
Confocal images (scale bar = 100 μm) of ALG beads with C-dots immobilised under 405 nm excitation for (a) ALG-C-dots-Ads and (d) ALG-C-dots-Hyb, respectively. Differences in fluorescent distribution result from varied C-dots diffusion kinetics within the bead system; (b and c) and (e and f) are each related multi-layer-scanned 3D images; (g) schematic figure shows the C-dots distribution resulting from their diffusion kinetics, fitted with three steps in the intra-particle model: (i) external diffusion (PFO), (ii) internal diffusion (PFO), and (iii) intra-particle pores filling (PSO).

Fig. 5a and d display the fluorescence distribution from single-layer scanning, while Fig. 5b, c, e, and f show the related 3D fluorescence distribution generated after multi-layer scanning. Although manual cutting of the wet beads during sample preparation may cause deformation, the fluorescence signal still provided valuable insights into the spatial distribution and immobilisation of C-dots within the alginate matrix.

Upon examining the particle distributions in the cross-sectional bead area after batch adsorption experiments, it was identified that C-dots were predominantly adsorbed in the outer layer of the beads. As shown in Fig. 5a, fluorescent particles are distributed from the outer layer to the inner part, with a thickness threshold measuring 100.04 μm. The density of signal spots shows a decreasing trend in the inner layers, with almost no visible fluorescence signals around a diameter of 223.35 μm. This observation is also evident in 3D images viewed from different angles (Fig. 5b and c), demonstrating that C-dots are distributed non-uniformly throughout the beads after adsorption/desorption. This validates that the adsorption of C-dots was primarily on the surface of the ALG-as-made beads, with diffusion into the beads occurring but with limited penetration depth and thus with decreasing fluorescence intensity. These findings are consistent with the fitted results of the adsorption kinetic models as well.

To further verify that the non-uniform distribution of C-dots is influenced by the interfacial processes during batch adsorption experiments, we pre-mixed C-dots with sodium alginate salt and then let the mixture cross-link to prepare the ALG-C-dots-Hyb hydrogel beads. The results, shown in Fig. 5d–f, indicate that beads exhibit a more uniform fluorescence distribution, with a diameter of around 887.48 μm and numerous smaller spots scattered throughout the images. Compared with the ALG-C-dots-Ads beads, which have a larger and more cohesive structure with concentrated high-intensity fluorescent signals at the surface, the ALG-C-dots-Hyb beads show a more homogeneously dispersed pattern with lower-intensity signals. Overall, fluorescence in ALG-C-dots-Ads is more localized, whereas in ALG-C-dots-Hyb, it is more widespread.

By measuring the fluorescence intensities of C-dots in the solution after immersing ALG-C-dots-Ads and ALG-C-dots-Hyb beads for the same duration (with identical C-dots content in both systems), we also observed that the solution containing ALG-C-dots-Hyb beads exhibited a notably weaker emission peak (Fig. S5†). This demonstrated that the adsorption of C-dots by ALG-as-made beads was limited due to their interconnecting layers structure with varying porosities. It also suggests that this morphology reduces the leakage of internal-loaded C-dots into the solution. This finding can help to tune the immobilisation and release processes of fluorescent particles for targeted bioimaging and biosensing applications.

We applied the adsorption kinetic models (PFO, PSO and intra-particle diffusion dynamics) to fit the adsorption capacity of the C-dots per unit ALG-as-made beads until they reached an equilibrium at the time *t*. The kinetic models were correlated with confocal microscopy visualisation to gain a deeper understanding of the mass transfer processes of C-dots in alginate hydrogel beads. The diffusion of the C-dots into the hydrogel beads (ALG-C-dots-Ads) is a three-step process, as depicted in Fig. 5g. The first step involves external diffusion, where adsorbate particles migrate across the liquid film surrounding the adsorbent. The initial high-concentration gradient drives rapid particle transport to the adsorbent surface due to the lower diffusion resistance within the boundary layer (Fig. 4c, indigo-shaded line). Subsequently, the decrease in the slope of the second stage (Fig. 4c, blue-shaded line) reflects increased diffusion resistance as surface adsorption sites become saturated, shifting the rate-limiting step to internal diffusion (*e.g.*, pore penetration). These initial two stages primarily describe the initial adsorption phase dominated by external mass transfer. The third stage (Fig. 4c, grey-shaded line), which has the lowest slope, corresponds to an equilibrium state in which the surface adsorption sites are fully saturated, and the driving force for further adsorption (*e.g.*, concentration gradients) diminishes significantly. Consequently, the transport of C-dots from the bulk liquid into the intra-particle pores becomes saturated. And the PFO model effectively describes the overall adsorption/desorption behaviour.^45^

The three-stage adsorption mechanism – (i) external diffusion, (ii) internal diffusion, and (iii) intra-particle pore filling – is visualized in confocal microscopy results. These findings highlight the critical role of bead morphology in diffusion kinetics and fluorescent particle distribution. Specifically, the concentrated fluorescent signal at the outermost layer of the bead cross-section suggests rapid surface-driven adsorption, with limited penetration into deeper pores due to steric or energetic barriers. This spatial heterogeneity underscores the need for C-dots immobilisation strategies (*e.g.*, surface functionalisation *vs.* bulk encapsulation) to align with bead architecture in order to optimise performance in applications such as fluorescence-based sensing, bioimaging, or controlled-release nanocomposites. It is worth mentioning that our general assumption is that diffusion kinetics should not impact the fluorescence signal, as confirmed by our confocal images and PL intensity analysis. In the absence of the hydrogel matrix and C-dots immobilisation, fluorescence signal quenching could be considered. However, the alginate hydrogel framework is expected to retain the fluorescence emission properties.

### 3.6 Possible adsorption mechanism and interactions

To correlate the theoretical models with the fluorescence distribution of C-dots within alginate beads, which reflect various steps, and to gain insights into how C-dots interact with the crosslinked alginate structure, thermoanalytical techniques and XPS were employed to identify possible interaction mechanisms.

Thermogravimetry plots of the freeze-dried ALG-as-made, ALG-C-dots-Hyb, and ALG-C-dots-Ads are given in Fig. S6a.† Upon heating under an inert environment (N_2_ (g)), all of the dried beads exhibited a three-stage mass loss. The first mass loss occurred between 30 °C and 200 °C, with ALG-as-made, ALG-C-dots-Hyb, and ALG-C-dots-Ads experiencing mass losses of 18.71%, 18.72%, and 21.22%, respectively. This loss is due to the evaporation of possible trapped moisture within the materials. Subsequently, the second and third mass losses occur between 200 °C and 800 °C, primarily due to the thermal decomposition of the alginate chains and numerous functional groups at increased temperatures. ALG-C-dots-Hyb and ALG-C-dots-Ads display similar trends and mass losses during this stage, with higher final weights remaining (34.90% and 37.14%, respectively) compared to ALG-as-made (27.76%). This indicates that the introduction of N-doped C-dots enhances the thermal stability of the alginate hydrogel system, which also proves the structural modifications of alginate.

DSC curves of ALG-as-made, ALG-C-dots-Ads, and ALG-C-dots-Hyb samples are shown in Fig. S6b.† Upon a heat–cool–heat cycle under an inert atmosphere (N_2_ (g)), all samples exhibited an endothermic trend up to 180 °C in the first heating scan, corresponding to the evaporation of physically adsorbed water and dehydration. The glass transition temperature observed from the second heating scan of ALG-as-made is 152 °C, which increases to 164 °C upon immobilisation of the C-dots on the alginates. This suggests that C-dots diffuse into the egg-box structure forming secondary bonds to enhance the chemical stability. Moreover, ALG-as-made exhibits a significant exothermic peak at 246 °C, indicating the typical degradation of the alginate backbone. In contrast, the C-dots-loaded samples exhibited multiple, less intense exothermic peaks, indicating delayed or reduced thermal degradation. This is attributed to the increased number density of C-dots, confirming enhanced thermal stability and stronger secondary interactions between the alginate and C-dots. These results correlate well with TGA data as described above, indicating that the incorporation of C-dots into alginate hydrogel beads, in turn, enhances their thermal stability and alters their thermal degradation behaviour, particularly in the hybrid form (ALG-C-dots-Hyb), contributing to higher thermal stability and more complex thermal degradation pathways.

The XPS spectra for ALG-as-made (I), ALG-C-dots-Hyb (II), and ALG-C-dots-Ads (III), in Fig. S7,† were used to investigate the adsorption mechanism and interactions between C-dots and alginate hydrogel beads prepared with different loadings methods.^23–25^ The presence of new peaks at the binding energy of 400.04 eV for C-dots-loaded samples in the fitted N 1s peaks (Fig. S7d†) confirmed the successful incorporation of C-dots in the alginate hydrogel matrix. In the fitted C 1s spectra (Fig. S7b†), the observed shifts in binding energies suggest interactions between the C-dots and the alginate matrix, particularly at the sites of oxygenated functional groups listed in Table S2.† These interactions, including surface adsorption and potential bonding between the C-dots and the matrix, enhance the overall stability and modify the properties of the hydrogel system. The combination of peak intensity shifts and binding energy changes confirms that the C-dots influence the chemical structure of the alginate hydrogel, particularly affecting the outer layers where adsorption occurs. Among all the peaks, a downward shift was observed exclusively for the C=O peaks, from 287.85 eV (I) to 287.75 eV (II) and 287.78 eV (III), suggesting that carbonyl groups may act as the most positively participating sites for interactions with the C-dots. This interaction likely involves binding or charge transfer with the C-dots, leading to a reduction in binding energy. This coordination with carbonyl groups was confirmed by the O 1s fitted spectra (Fig. S7c†). As shown in Table S2,† after immobilisation of the C-dots, the C=O peaks shifted slightly to 531.35 eV (II) and 531.01 eV (III), suggesting possible weak bonding interactions or charge transfer. Furthermore, the slight shifts of the C–OH peak suggest that the chemical interfaces between the C-dots and the alginate matrix could involve hydrogen bonding or electrostatic forces. The decreased intensities in the C=O and C–OH peaks for the alginate hydrogels with C-dots loading confirmed such interactions. Overall, the XPS results confirmed that oxygenated functional groups, particularly carbonyl and hydroxyl groups within the alginate structure, contribute to bonding with C-dots. This interaction enhances thermal stability and leads to complex thermal transitions, aligning with the findings from thermoanalytical analyses.

## 4. Conclusions

In conclusion, fluorescent N-doped C-dots were prepared using a hydrothermal method, exhibiting blue emission which is excitation-independent at 435 nm. Alginate hydrogel beads were prepared using a dropwise extrusion method and cross-linked with Ca^2+^ to form beads with mesopores, suggesting selective adsorption capabilities. Batch adsorption experiments were performed to study the diffusion and distribution behaviour of the synthesised C-dots within the fabricated hydrogel matrix. The adsorption kinetics align well with the pseudo-first-order model, while the intra-particle diffusion model exhibits three linear fits, indicating the mass transfer processes of C-dots in alginate hydrogel beads. Among them, the concentration-dependent diffusion of fluorescent particles from the external solution to the surface of hydrogel beads and binding with active sites are rapid steps, while diffusion to the interconnecting layers is the rate-determining step. The three stages of diffusion demonstrate the impact of crosslink-induced porous structure on the kinetics and distribution of fluorescent particles. Confocal microscopy images confirmed the distribution of fluorescent particles within the alginate matrix. After (i) batch adsorption experiments, C-dots were primarily localized on the surface with limited depth penetration and uneven distribution, whereas (ii) the premixed composites prior to cross-linking showed a more homogeneously dispersed pattern. Both manufacturing methods demonstrate the effective immobilisation properties of alginate hydrogel beads for fluorescent particles. The product exhibits a stable fluorescence response, and limited C-dots leach from the matrix due to the interconnected layer structure with varying porosities. Mechanism studies confirm that carbonyl and hydroxyl groups in the alginate structure contribute to bonding with C-dots, enhancing thermal stability and leading to complex thermal transitions, which reflect improved structural integrity and interactions. Overall, these studies highlight the importance of C-dots distribution and the method they are applied to a matrix prior to imaging. Consequently, in imaging or drug delivery studies involving the immobilisation of C-dots within complex network structures, it is necessary to first optimise their uptake into the hydrogel structure to achieve the most reliable results in imaging, as well as to understand the diffusion limitations and the extent of particle migration within the carrier. Considering these key points will potentially help to improve manufacturing processes for targeted functional applications.

## Data availability

The authors confirm that the data supporting the findings of this study are available within the article [and/or] its ESI.†

## Author contributions

Jingyi Wang: conceptualization, data curation, formal analysis, investigation, methodology, validation, visualization, writing – original draft, writing – review & editing. Luz Carime Gil Herrera: conceptualization, preliminary data curation, investigation, writing – original draft. Ozge Akbulut: conceptualization, funding acquisition, project administration, writing – review & editing. Ahu Gümrah Dumanli: conceptualization, formal analysis, validation, funding acquisition, project administration, resources, supervision, visualization, writing – review & editing.

## Conflicts of interest

There are no conflicts to declare.

## Supplementary Material

RA-015-D5RA01045D-s001
