# Supplementary material for: Investigating the fluorescence in C-dots immobilised on alginate hydrogels-a study on diffusion kinetics and adsorption mechanisms
Source: RSC Adv. 2025 May 15;15(21):16281–91. doi: 10.1039/d5ra01045d (PMC12080466; doi:10.1039/d5ra01045d)
Supplement: RA-015-D5RA01045D-s001 [file RA-015-D5RA01045D-s001.pdf]

Supplementary Information (SI) for RSC Advances.
This journal is © The Royal Society of Chemistry 2025

Supporting Information

Investigating the Fluorescence in C-Dots immobilized on Alginate Hydrogels-

A study on Diffusion Kinetics and Adsorption Mechanisms

Jingyi Wang,[a,b] Luz Carime Gil Herrera,[a,b] Ozge Akbulut,[c] and Ahu Gümrah Dumanli *[a,b]

a *Department of Materials, The University of Manchester,*
Oxford Road, Manchester M13 9PL, U.K.
b Henry Royce Institute, The University of Manchester,
Oxford Road, Manchester M13 9PL, U.K.
c Faculty of Engineering and Natural Sciences, Sabanci University,
Istanbul 34956, Turkiye.

** Corresponding author:* ahugumrah.parry@manchester.ac.uk

Supporting Figures

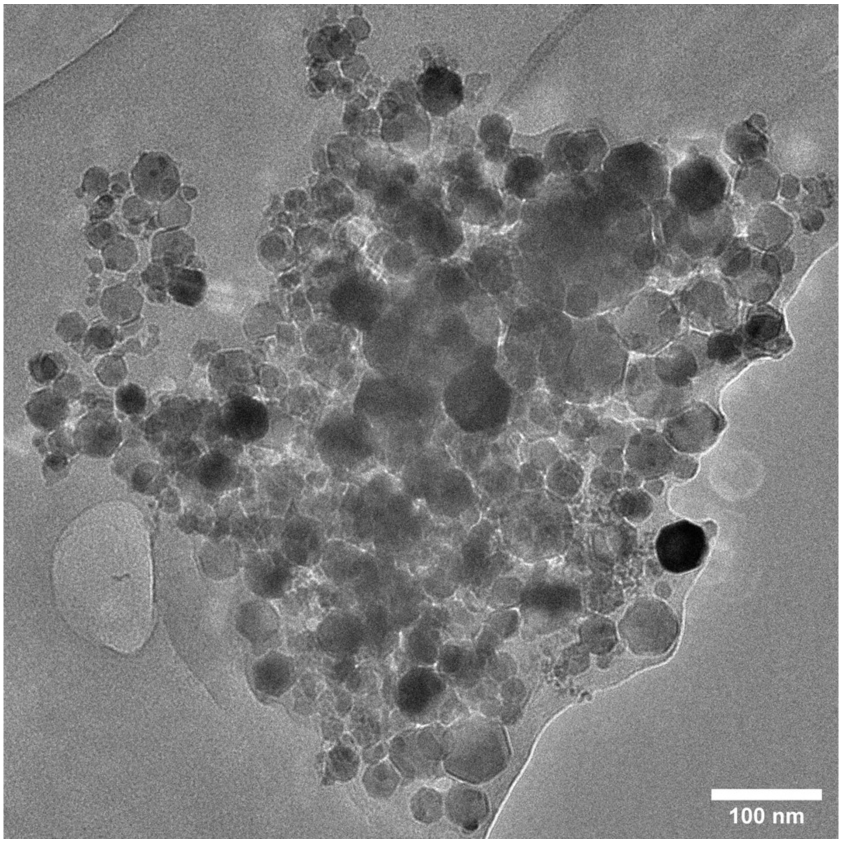

Figure S1. Transmission electron microscopy (TEM) image of synthesized C-dots with a 100 nm scale bar, where the size and size distribution of the synthesized C-dots were illustrated and analyzed.

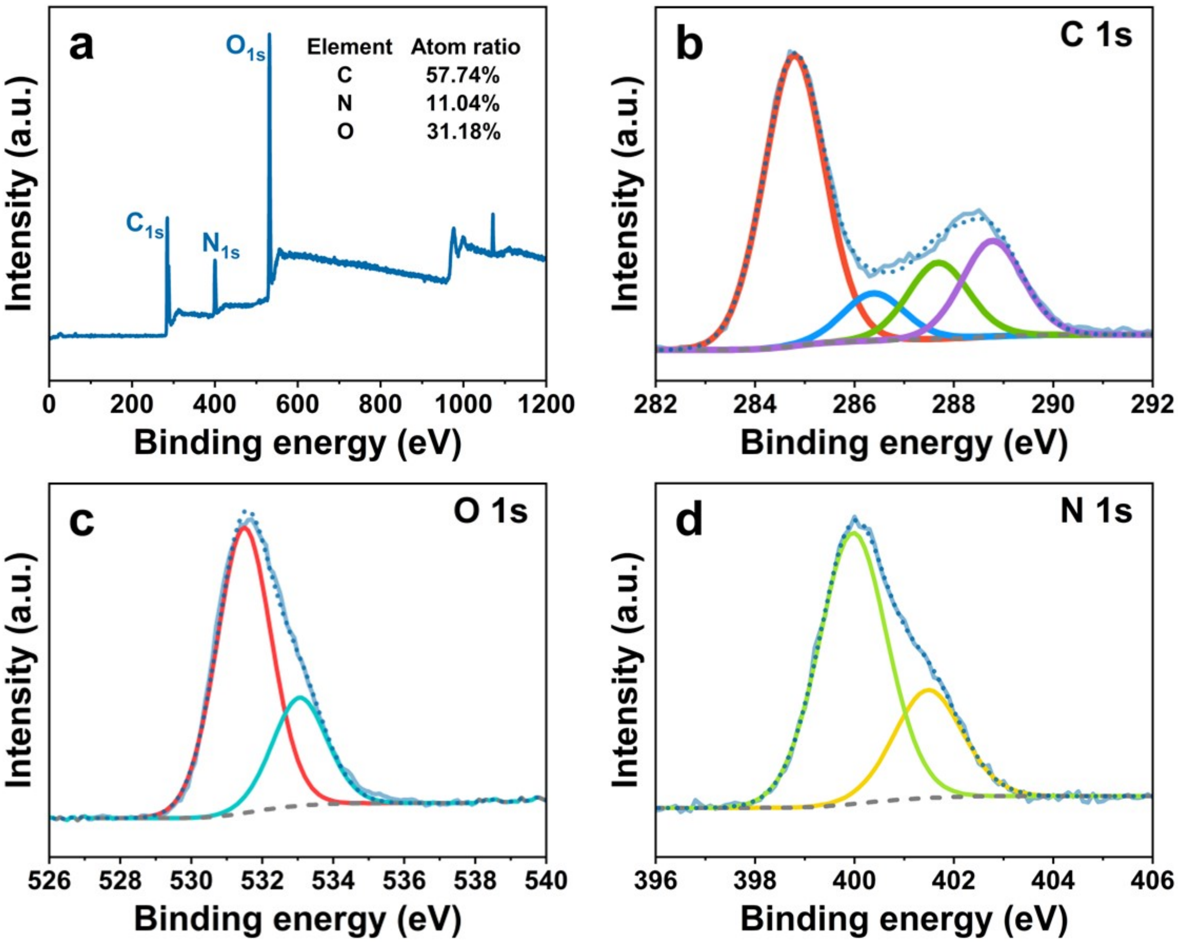

Figure S2. **(a)** XPS survey spectra, **(b)** C 1s spectra, **(c)** O 1s spectra, and **(d)** N 1s spectra of synthesized N-doped C-dots.

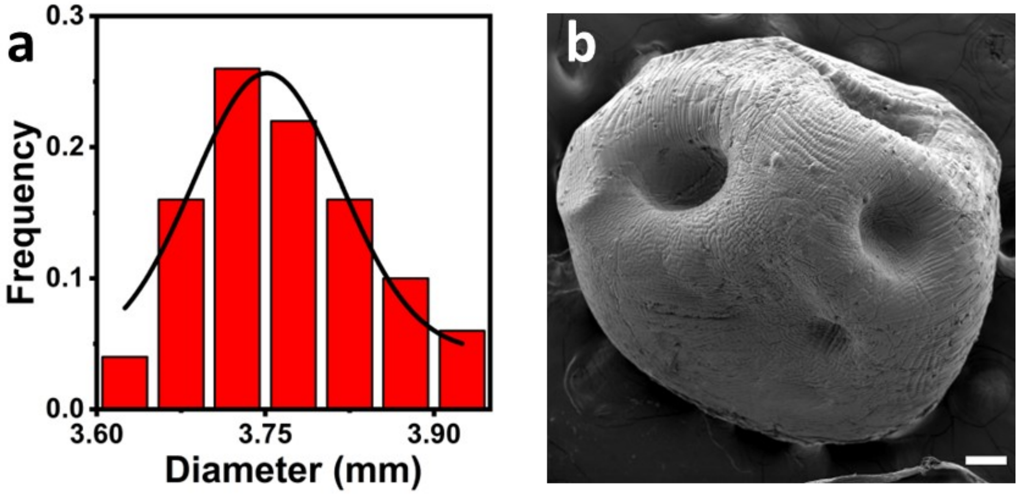

Figure S3. (a) Size distribution analysis of ALG-as-made hydrogel beads based on their optical microscopy images, and **(b)** SEM image of the surface of a freeze-dried ALG-as-made bead, scale bar = 100 μm.

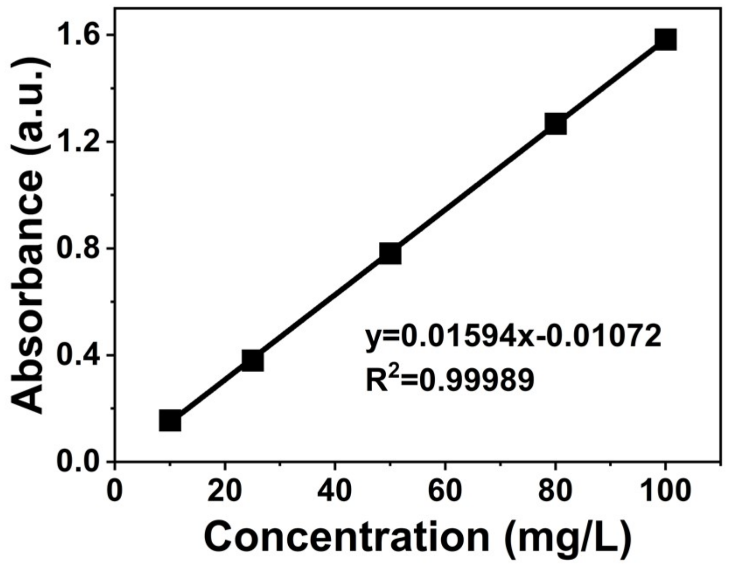

Figure S4. Concentration calibration plot from UV-Vis absorption of C-dots solution with concentrations of 100, 80, 50, 20, and 10 mg/L, respectively. All samples were measured at neutral pH.

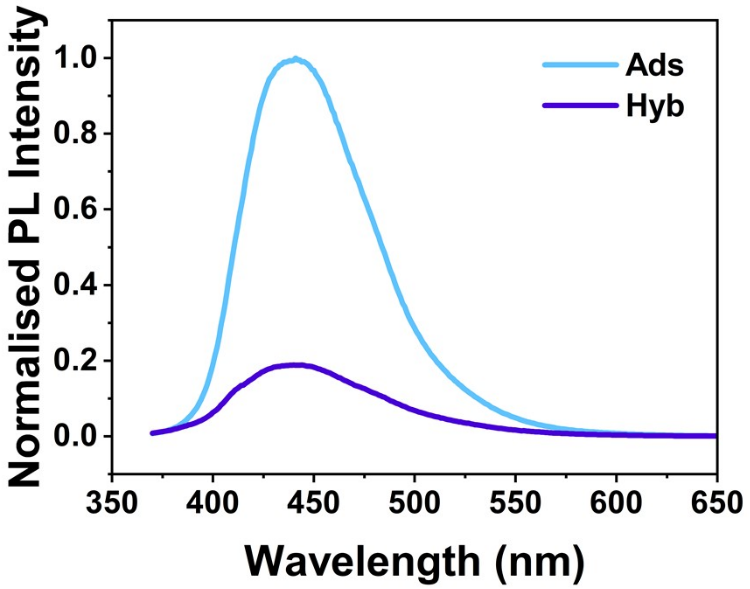

Figure S5. Normalised PL intensity of C-dots in the solution outside the beads after immersing ALG-C-dots-Ads and ALG-C-dots-Hyb for the same duration, with identical C-dot content in both systems.

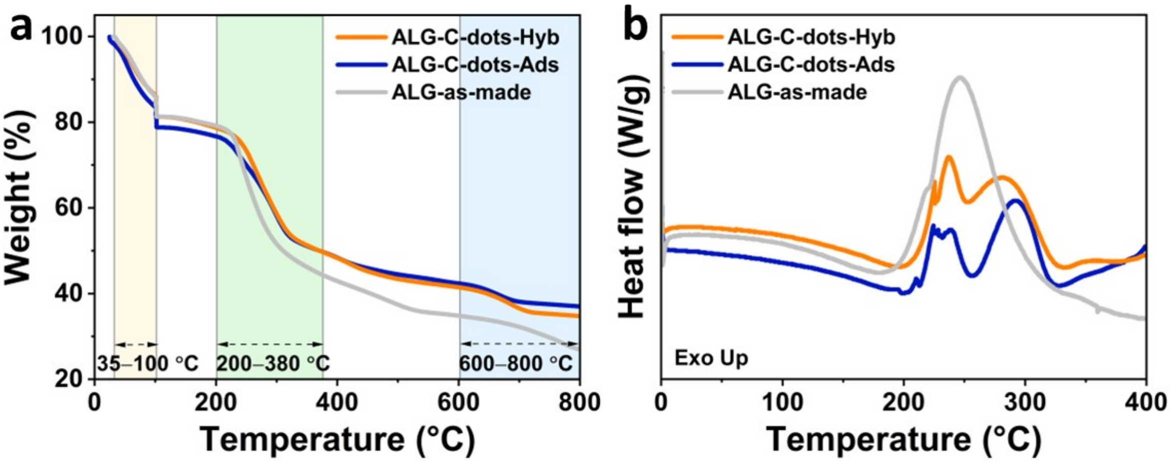

Figure S6. (a) TGA and **(b)** DSC analysis curves of ALG-as-made, ALG-C-dots-Hyb, and ALG-C-dots-Ads beads, respectively.

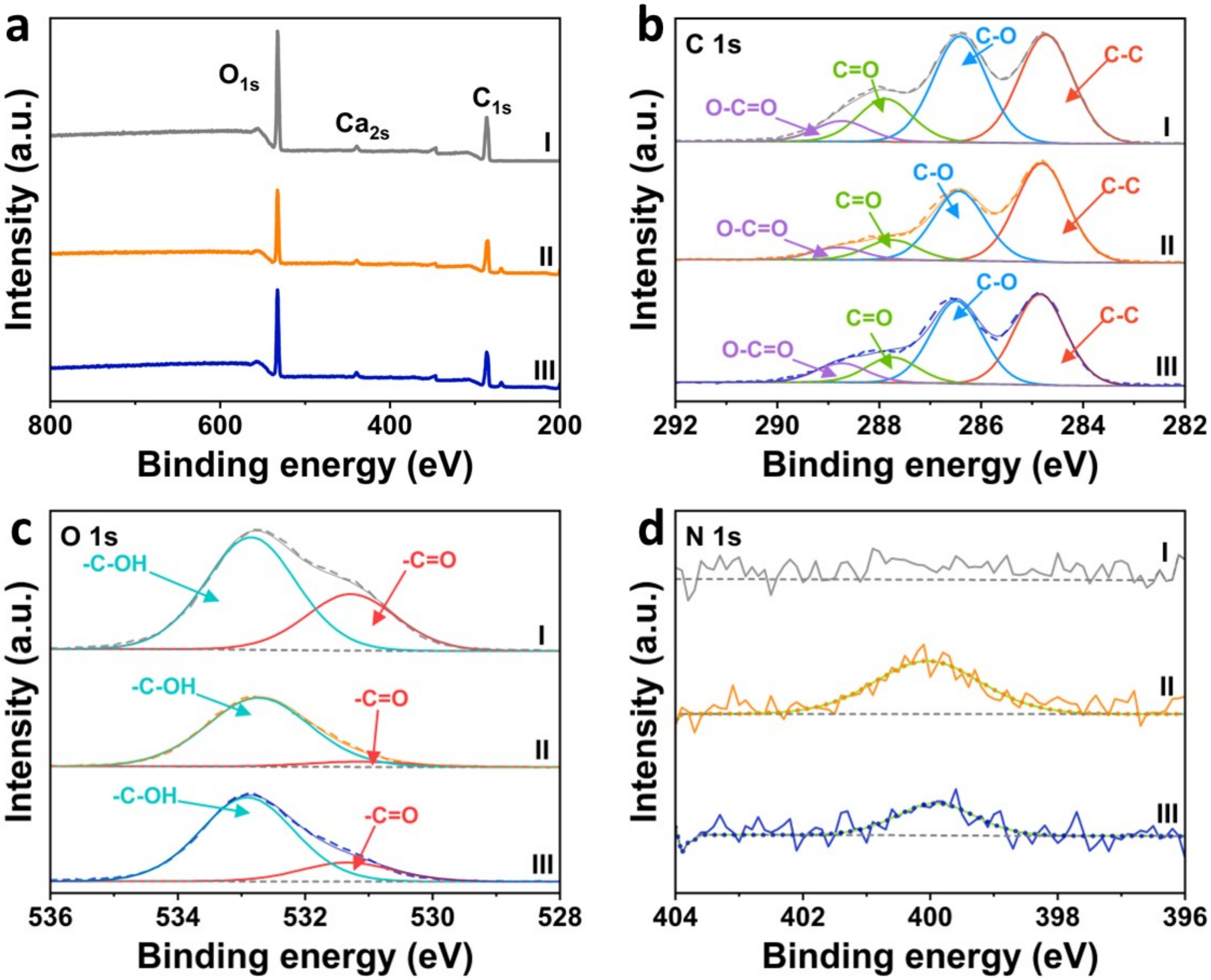

Figure S7. (a) XPS survey spectra, **(b)** C 1s spectra, **(c)** O 1s spectra, and **(d)** N 1s spectra of freeze dried ALG-as-made, ALG-C-dots-Hyb, and ALG-C-dots-Ads bead samples, respectively.

Supporting Tables

Table S1. Kinetic parameters for C-dots adsorption with 4 batches of initial concentrations on ALG-as-made beads.†

Concentration (mg/L)	$q_{e,exp}$ (mg/g)	Pseudo-first-order model			Pseudo-second-order model		
		$q_{e,cal}$ (mg/g)	K_1 (/min)	R^2	$q_{e,cal}$ (mg/g)	K_2 (g/mg min)	R^2
100	0.8543	0.8778	0.0166	0.930	0.9388	0.0230	0.859
80	0.6918	0.7003	0.0159	0.900	0.7513	0.0269	0.839
50	0.4236	0.4266	0.0249	0.900	0.4598	0.0773	0.824
20	0.2186	0.2178	0.0224	0.931	0.2305	0.1292	0.873

† $q_{e,exp}$ (mg/g) is the experimental value of adsorption capacity, $q_{e,cal}$ (mg/g) is the theoretical value of adsorption capacity.

Table S2. XPS peak binding energy shifts for ALG-as-made, ALG-C-dots-Hyb, and ALG-C-dots-Ads bead samples, respectively.

**C 1S**	C-C (eV)	C-O (eV)	C=O (eV)	O-C=O (eV)
ALG-as-made (I)	284.74	286.40	287.85	288.74
ALG-C-dots-Hyb (II)	284.84	286.49	287.75	288.78
ALG-C-dots-Ads (III)	284.82	286.44	287.78	288.81

**O 1S**	C=O (eV)	C-OH (eV)
ALG-as-made (I)	531.28	532.87
ALG-C-dots-Hyb (II)	531.35	532.92
ALG-C-dots-Ads (III)	531.01	532.73
